# Keys to Achieving Target First Medical Contact to Balloon Times and Bypassing Emergency Department More Important Than Distance

**DOI:** 10.1155/2018/2951860

**Published:** 2018-05-21

**Authors:** Saad Ezad, Allan J. Davies, Hooria Cheema, Trent Williams, James Leitch

**Affiliations:** John Hunter Hospital, Hunter New England Health, Newcastle, NSW, Australia

## Abstract

**Background:**

Australian guidelines advocate primary percutaneous coronary intervention (PPCI) as the reperfusion strategy of choice for ST elevation myocardial infarction (STEMI) in patients in whom it can be performed within 90 minutes of first medical contact; otherwise, fibrinolytic therapy is preferred. In a large health district, the reperfusion strategy is often chosen in the prehospital setting. We sought to identify a distance from a PCI centre, which made it unlikely first medical contact to balloon time (FMCTB) of less than 90 minutes could be achieved in the Hunter New England health district and to identify causes of delay in patients who were triaged to a PPCI strategy.

**Methods and Results:**

We studied 116 patients presenting via the ambulance service with STEMI from January 2016 to December 2016. In patients who were taken directly to the cardiac catheterisation lab, a maximum distance of 50 km from hospital resulted in 75% of patients receiving PCI within 90 minutes and approximately 95% of patients receiving PCI within 120 minutes. Patients who bypassed the emergency department (ED) were significantly more likely to have FMCTB of less than 90 minutes (*p* < 0.001) despite having a longer travel distance (28.5 km versus 17.4 km, *p* < 0.001). Patients transiting via the ED were significantly more likely to present out of hours (60 versus 24.2% *p* < 0.001).

**Conclusions:**

Patients who do not bypass the ED have a longer FMCTB across all spectrum of distances from the PCI centre; therefore, bypassing the ED is key to achieving target FMCTB times. Using a cutoff distance of 50 km may reduce human error in estimating travel time to our PCI centre and thereby identifying patients who should receive prehospital thrombolysis.

## 1. Introduction

Recently published Australasian guidelines advocate primary percutaneous coronary intervention (PPCI) as the reperfusion strategy of choice for ST elevation myocardial infarction (STEMI) in patients in whom it can be performed within 90 minutes of first medical contact; otherwise, fibrinolytic therapy is preferred [1]. The Hunter New England health district covers an area of 130,000 square kilometres served by a single PPCI centre. The prehospital triage of patients in the Hunter region presenting with STEMI to the New South Wales Ambulance Service (NSWAS) has been described in detail previously [2]. Briefly, paramedics record a 12-lead electrocardiogram for all patients with symptoms suggestive of myocardial infarction. If this is interpreted as a STEMI by the Glasgow algorithm, it is transmitted to the on-call cardiology team at the John Hunter Hospital (JHH). Following discussion with an interventional cardiologist, the most appropriate reperfusion strategy is chosen, either prehospital thrombolysis or primary PCI. Delays in the first medical contact to balloon (FMCTB) time have been associated with increased mortality and left ventricular dysfunction [3]. Determining the optimal reperfusion strategy in a large health district is a challenge for the paramedics and cardiologists alike. The objective of this study was to identify a distance from hospital where it is very unlikely that the patient will receive primary PCI in a timely fashion, therefore enabling paramedics to proceed with prehospital thrombolysis. Our second objective was to identify causes of delays in the FMCTB time in patients in whom primary PCI was the chosen reperfusion strategy.

## 2. Methods

Data were prospectively collected into a departmental database for all patients presenting to JHH for PPCI from 1 January 2016 to 31 December 2016. Patients were eligible for inclusion in this study if they presented via the New South Wales Ambulance Service (NSWAS) to either the emergency department (ED) or directly to the cardiac catheterisation laboratory with the diagnosis of STEMI. Patients who self-presented to the emergency department were excluded, along with those from peripheral hospitals who were transferred for rescue PCI. Patients were stratified by a FMCTB of either less than or greater than 90 minutes and according to whether they presented directly to the cardiac catheterisation laboratory or via the emergency department. First medical contact time (FMC) was defined as first direct contact by a health practitioner with the patient. This is consistent with the current European Society of Cardiology 2017 guideline “Acute myocardial infarction in patients presenting with ST-segment elevation myocardial infarction” [4]. We specified an appropriate distance as that at which 75% of patients would have an FMC to balloon time of less than 90 minutes if they were transported directly to the catheterisation lab as per current guideline recommendations. We arbitrarily defined a long prehospital time as time from FMC to arrival at PCI hospital greater than 60 minutes. To identify outliers in the patient sample, we defined a long Emergency Department time as being greater than 30 minutes and a long catheterisation laboratory arrival to balloon time as being greater than 30 minutes. Patients who had incomplete ambulance records were excluded from the study as first medical contact time could not be accurately identified. Procedural notes and cardiac catheterisation images were reviewed to identify time of reperfusion (balloon inflation) and cases of complex PCI (defined as use of buddy wire or guideliner to facilitate balloon or stent delivery).

All statistical analyses were conducted using Stata version 14 (Stata Corp).

## 3. Results

We identified a total of 116 patients who underwent PPCI and had prehospital assessment by the NSWAS in 2016.

Baseline characteristics of patients who transferred directly to the catheterisation lab compared with those who transited via the emergency department are shown in Table 1. There were no significant differences in baseline characteristics, except mean serum creatinine on admission was higher in the group of patients taken to the catheterisation lab via the emergency department.

For the total cohort, the mean distance from the site of first medical contact to the cardiac catheterisation lab was 23.8 km, and the median FMCTB time was 91.5 minutes (IQR 79–117.5 minutes). The median time spent in the ambulance was 47 minutes (IQR 35–59 minutes). The maximum travelled distance was 78 km, and the minimum distance was 2 km. A total of 12 patients travelled longer than 50 km. In patients taken directly to the cardiac catheterisation lab, a maximum distance of 50 km from hospital resulted in 75% of patients receiving PCI within 90 minutes and approximately 95% of patients receiving PCI within 120 minutes (Figure 2).

**Figure 1:**
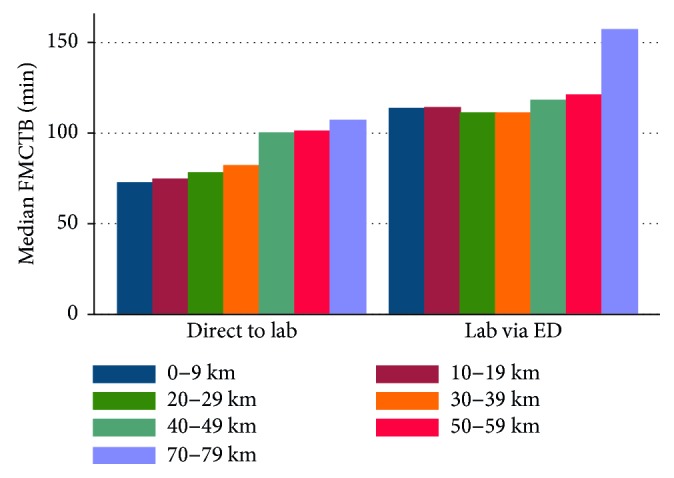
Comparison of median FMC to balloon times by stratum of distance, grouped according to whether the patient presented directly to the cardiac catheterisation lab or via the emergency department.

**Figure 2:**
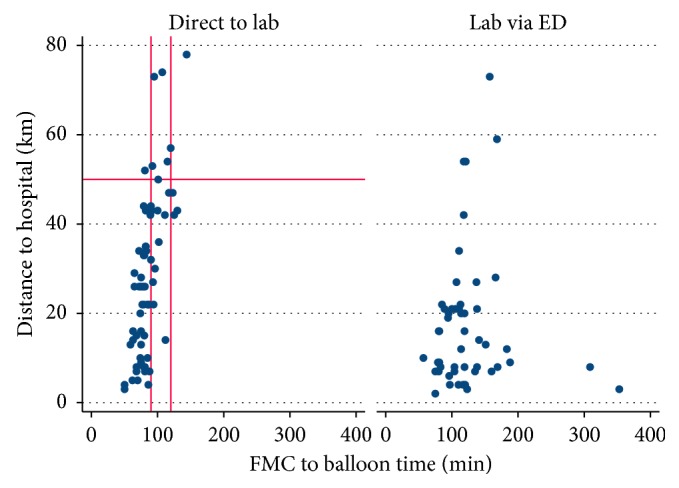
Scatter plot of distance from hospital and FMC to balloon times, grouped according to whether the patient presented directly to the cardiac catheterisation lab or via the emergency department.

Throughout all stratum of distance, FMCTB times were shorter among patients presenting directly to the cardiac catheterisation lab (Figure 1). There was much more homogeneity in FMCTB times in patients taken directly to the catheterisation lab compared to those taken via the emergency department (Figure 2).

Procedural characteristics are presented in Table 2. Patients who were taken directly to the catheterisation laboratory, bypassing the emergency department, were significantly more likely to have FMCTB time of less than 90 minutes (*p* < 0.001). The median travel distance was significantly longer for patients presenting directly to the cardiac catheterisation lab compared to those presenting via the emergency department (28.5 km versus 17.4 km, *p* < 0.001).

For those patients who presented via the emergency department, the median time spent in the emergency department was 19 minutes. Out of the 50 patients who transited through the emergency department, 33 patients spent longer than 30 minutes waiting for transfer to the catheterisation laboratory. 16 patients (32%) had clinical reasons meaning they could not bypass the ED. The most common reason was ECG changes that initially did not meet Glasgow criteria resulting in review in the ED as opposed to direct catheterisation lab transfer (12 patients). A total of four patients required CT scanning of the aorta or brain prior to catheterisation, and another two required intubation and mechanical ventilation prior to catheterisation, whilst four patients were delayed due to the cardiac catheter lab being occupied. No clear reason for delay was identified in 10 patients.

Once a patient had arrived in the catheterisation laboratory, time to balloon was significantly affected by complexity of PCI, patients requiring complex PCI having a mean lab arrival to balloon time of 35.6 minutes compared with 23.2 minutes in noncomplex PCI (*p* < 0.001).

## 4. Discussion

Our study is the first to demonstrate the effect of distance from a PCI centre and bypassing the ED on FMCTB times in a large Australian health district. We have shown that bypassing the ED significantly increases the likelihood of achieving a FMCTB of less than 90 minutes. Bypassing the ED reduced the FMCTB time by 32.5 minutes in concordance with previous studies [5, 6], despite patients having on average travelled longer distances (28.5 versus 17.4 km *p* < 0.001). Patients who live a greater distance from the PCI centre can still achieve a FMCTB time of less than 90 minutes if the catheterisation lab team is mobilised and ready to receive the patient, thereby bypassing the emergency department, similar to previous findings in the Netherlands [7].

A key barrier to increasing the number of patients who bypass the ED appears to be a higher proportion of out of hours cases presenting via the ED (60 versus 24.2%; *p* < 0.001); this is likely due to the PCI team being off site and the delay in arrival meaning the patient cannot be transferred directly to the catheterisation lab. These findings highlight the challenge of achieving FMCTB targets in patients who live a short distance from a PCI centre relates to their in-hospital care, not their prehospital care. The systems of care currently in place for prehospital cardiac catheterisation lab activation work very well when the catheterisation lab team arrives either before or at the same time as the ambulance as patients can be transported directly to the lab. We have demonstrated that improvements in the systems of care for patients arriving prior to the catheterisation lab team need to be made to improve FMCTB times. One proposed change could involve having a dedicated team of health care professionals (emergency doctor and nurse) on site who are trained to escort the patient to the catheterisation laboratory and begin the initial setup whilst awaiting arrival of the catheterisation lab team.

Bypassing the ED has also been associated with improved clinical outcomes including lower mortality [8–11] and improved ejection fraction [12]. This study highlights the importance of bypassing the emergency department to reduce the time to reperfusion and consolidates on knowledge gained from previous studies on the importance of bypassing the emergency department [6, 13].

However, not all patients may safely bypass the ED, with 16 patients (32%) in this study having clinical reasons for ED evaluation. Twelve of these patients had ECGs which did not trigger the Glasgow algorithm, and therefore, there was no prehospital activation of the catheterisation lab. Once a diagnostic ECG is obtained, ED staff could potentially transfer the patient to the catheterisation lab as described above to reduce time to balloon inflation. 4 patients (25%) underwent computed topography (CT) imaging of either the aorta or the brain. A study by Armstrong et al. found out of 45 CT scans performed on patients with STEMI where the lab was activated, only 2 (4%) led to catheterisation not being performed [14]. The authors went on to comment that the clinical presentation of these 2 patients was more consistent with significant intracerebral pathology. These findings suggest careful consideration should be given as to whom requires a CT prior to catheterisation and in cases where significant diagnostic uncertainty exists or the potential to do harm with the use of antiplatelet agents such as in suspected intracerebral haemorrhage, CT imaging should be expedited to facilitate a rapid transfer to the catheterisation lab if appropriate.

Prehospital thrombolysis with rescue PCI if required has been demonstrated to be a safe and efficacious strategy for reperfusion in geographically remote regions of Australia [2]. Currently, we rely on paramedics and the interventionalists' knowledge of local geography and traffic conditions to estimate the prehospital time. Our study demonstrates that a distance of over 50 km from the PCI centre results in a 75% chance of achieving a FMCTB time of less than 90 minutes, if the patient is taken directly to the cardiac catheterisation lab. This provides important local knowledge which can help reduce human error in estimating prehospital transfer time, resulting in more accurate decisions regarding the most appropriate reperfusion strategy to be made.

This study has several limitations to consider; firstly, it is a one-year snapshot of the relationship between our health service and the emergency medical services. Sampling from a longer time period may provide more accurate results; however, given the recent changes in the transport infrastructure resulting in shorter transit times from the Hunter region, we wanted to use a contemporary cohort of patients. Traffic and weather conditions are unpredictable which makes interpretation of a cutoff distance from the PCI centre for PPCI reperfusion strategy less clear-cut. Furthermore, the Hunter region has a well-established prehospital triage system which may not be the case in other health districts, meaning these findings may not be generalizable to such regions.

## 5. Conclusions

We have demonstrated in a large, geographically challenging health district bypassing the ED is key to achieving FMCTB times of less than 90 minutes. As a greater proportion of out-of-hours cases transfer via the ED, efforts need to be made to ensure the catheterisation lab is activated as early as possible or ED staff are trained to initiate initial treatment in the catheterisation lab in order to facilitate a direct transfer from the ambulance. A distance of greater than 50 km makes it unlikely a FMCTB time of less than 90 minutes can be achieved, and in these patients prehospital thrombolysis with transfer to a PCI-capable centre should be preferred.

## Figures and Tables

**Table 1:** Baseline characteristics.

Variable	Group “direct” (*n*=66)	Group “ED” (*n*=50)	*p* value
Age	64.5 (11.5)	63.7 (11.6)	0.73
Male sex	55 (83.3%)	41 (80.4%)	0.17
SBP	126 (22.2)	129 (21.8)	0.44
BMI	30.0 (4.7)	29.5 (4.0)	0.53
Prior MI	13 (19.7%)	6 (11.8%)	0.25
Prior CABG	3 (4.5%)	4 (7.8%)	0.46
Prior PCI	10 (15.2%)	5 (9.8%)	0.39
Diabetes	16 (24.2%)	10 (19.6%)	0.55
Hypertension	37 (56.1%)	29 (56.9%)	0.93
Dyslipidemia	27 (40.9%)	20 (39.2%)	0.85
Smoking history	47 (71.2%)	32 (62.8%)	0.33
Baseline creatinine	83 (22.8)	96 (38.7)	0.04

**Table 2:** Procedural characteristics.

Variable	Group “direct” (*n*=66)	Group “ED” (*n*=50)	*p* value
FMC to balloon time (min)	81.5 (75–93)	114 (95–138)	<0.001
Travel distance (km)	28.5 (18.1)	17.4 (15.3)	<0.001
Prehospital time (min)	48 (40–63)	41.5 (31–54)	0.009
Weekday case	57 (86.4%)	37 (74.0%)	0.09
After hours case	16 (24.2%)	30 (60%)	<0.001
Prehospital cardiac arrest	4 (6%)	4 (8%)	0.68
Peak troponin	63733 (69667)	55939 (55957)	0.53
In-hospital death	4 (6%)	4 (8%)	0.71
30-day readmission	5 (7.6%)	7 (13.7%)	0.28
30-day mortality	5 (7.6%)	5 (10%)	0.68

## Data Availability

Data can be requested on an individual case basis via the corresponding author.
